# Sustainability of Funding for HIV Treatment Services: A Cross-Sectional Survey of Patients' Willingness to Pay for Treatment Services in Nigeria

**DOI:** 10.9745/GHSP-D-21-00550

**Published:** 2022-04-28

**Authors:** Olawale Durosinmi-Etti, Bruce Fried, Karine Dubé, Sean Sylvia, Sandra Greene, Akudo Ikpeazu, Emmanuel Kelechi Nwala

**Affiliations:** a Health Leadership Program, Gillings School of Global Public Health, University of North Carolina at Chapel Hill, NC, USA.; b National AIDS and Sexually Transmitted Illnesses Control Program, Federal Ministry of Health, Abuja, Nigeria.; c John Snow Inc., Abuja, Nigeria.

## Abstract

**Background:**

About 50% of individuals needing HIV treatment are unable to access required services primarily due to the inability of the Nigerian HIV treatment program to meet patient needs. We explored patient willingness to pay for HIV treatment, which can inform the feasibility of cost recovery through patient fees to contribute to the funding of HIV treatment services in Nigeria.

**Methods:**

We conducted a cross-sectional survey of 400 people living with HIV randomly selected from 15 health care facilities providing free HIV treatment services in 2 medium and high HIV burden states (Lagos, Enugu) and the Federal Capital Territory Abuja. We calculated the elasticity of the price that patients were willing to pay per month relative to the estimated current cost of providing HIV treatment services per patient and determined the patient coverage and potential cost recovery at each price point.

**Results:**

We found that 92% of patients were willing to pay for HIV treatment. The mean amount patients were willing to pay was 3,000 naira (US$7.50) per month with about 18% of patients willing to pay the current monthly price of 5000 naira (US$12.50). The availability of financial support from family and friends (odds ratio [OR]=14.209; *P*=.001; 95% confidence interval [CI]=0.151, 0.285), lack of employment (OR=0.190; *P*=.02; 95% CI=0.015, 0.202), monthly income (OR=2.476; *P*<.001; 95% CI=84.698, 737.233), and change in monthly income (OR=2.015; *P*<.001; 95% CI=0.003, 0.229) were associated with willingness to pay.

**Conclusion:**

Many Nigerian patients are willing to contribute to funding for HIV treatment and this can enhance domestic funding for HIV treatment and equitable access to treatment through proper segmentation of patients based on willingness and capacity to pay. Measures must be put in place to reduce the cost of accessing HIV treatment and promote financial empowerment of people living with HIV to improve willingness to pay for treatment.

## Abstract (precis)

Many Nigerian patients are willing to pay for HIV treatment, but several socioeconomic factors play significant roles in willingness and capacity to pay for treatment and the maximum amounts patients are willing to pay.

## Teaser

**Key Findings:**

- Most people living with HIV (PLHIV) receiving free HIV treatment services are willing to pay for the services if such free treatment services are no longer available.
- The socioeconomic status of PLHIV plays a critical role in the willingness to pay for treatment. The willingness is further enhanced by the availability of financial support from friends and family.
- The maximum price that most patients are willing to pay for HIV treatment services is less than the current cost of providing services and the different price points are elastic relative to the estimated current price of providing services.

**Key Implications:**

- The domestic funding for HIV treatment services in low-income countries can be improved through cost recovery from patient contribution to the cost of treatment.
- Equitable access to HIV treatment services can be achieved by ensuring that patients are segmented based on socioeconomic status to identify those that can benefit from free services, subsidized and full-priced payment for treatment services.
- Willingness to pay for HIV treatment services and funding sustainability of the HIV treatment program can be enhanced by reducing the cost of treatment, which improves treatment affordability.

## BACKGROUND

Nigeria is among the countries with the highest number of people living with HIV (PLHIV) in the world.^1^ The country's 1.4% HIV prevalence in the adult population is considered low, but due to the large population of more than 180 million people, about 1.9 million people were living with HIV in Nigeria in 2019.^1^ Despite recording a 13% reduction in new HIV infection in the last 10 years, about 53,000 and 45,000 deaths were reported from HIV-related illnesses in 2018 and 2019, respectively.^2^ Data also indicate that Nigeria accounted for 2 of every 3 new HIV infections in west and central Africa in 2019.^2^

In 2011, the United Nations political declaration on HIV/AIDS called on the international community to mobilize between US$22–24 billion for the global response to HIV/AIDS in low- and middle-income countries by 2015; however, this target was not achieved, as only about US$19.1 billion was made available by the end of 2016.^3^ Based on UNAIDS estimates, a new funding target indicated that about US$26.2 billion will be required to enable the world to be on course to end HIV/AIDS as a public health threat in low- and middle-income countries by 2030.^4^ Therefore, the currently available resources must increase by US$1.5 billion annually from 2016 to enable the funding target of 2020 to be achieved.^5^

### Context of HIV/AIDS Funding in Nigeria

A review of national expenditures on HIV in Nigeria between 2009 and 2016 indicates that international funding from the United States President's Emergency Plan for AIDS Relief (PEPFAR) and the Global Fund accounted for about 88% of total funding for HIV in Nigeria.^6^ The government of Nigeria, private philanthropy, and the private sector, including out-of-pocket payments, provided the remainder of the funding for HIV services. Current data show that of the estimated 1.8 million PLHIV in Nigeria, 1.4 million (78%) have access to lifesaving antiretroviral therapy (ART) resulting in a treatment gap of 400,000 million (22%) PLHIV.^7^ The current challenge with ART coverage is with poor health-seeking behavior and poor uptake of services, particularly among key populations, pregnant women, and children. In Nigeria, limited access to treatment is due primarily to the lack of financial capacity of the HIV treatment program in Nigeria to provide ART and laboratory monitoring for those who need these services.^8^^,^^9^ According to the National Agency for the Control of AIDS (NACA), international funding for HIV response in Nigeria was about US$255 million in 2007.^6^ This increased over the years to about US$700 million in 2013 followed by a dip to just below US$450 million in 2014. Similarly, the Government of Nigeria increased its funding from about US$44 million in 2007 to slightly more than US$171 million in 2014. Due to huge funding gaps between availability and need, donor funding is now prioritized to ensure that clients who are already on treatment have unhindered access to treatment, rather than spending such funds for widespread HIV screening in those who do not know their HIV status which will increase the number of new patients who will require treatment in the future.

In Nigeria, limited access to treatment is due primarily to the lack of financial capacity of the HIV treatment program to provide ART and laboratory monitoring for those who need these services.

Based on the estimated gap between the number of people receiving treatment for HIV and the total number of individuals who require treatment, about 1 million additional patients require treatment from 2020.^8^ The average cost of accessing antiretroviral (ARV) drugs alone is US$96 per year per patient (US$8 per month), while the average cost of HIV treatment services (including ARVs and laboratory services and staff salary) is estimated at US$140 per year per patient.^6^ Current estimates indicate that an additional US$58 million will be required annually to add about 440,000 PLHIV into free treatment services as planned by the Nigerian government.^6^

Due to the chronic nature of HIV infection, there are long-term implications for treatment cost as treatment must last for the entire life of the patient in the absence of a cure. In addition, the treatment cost increases as patients switch from a first-line treatment regimen—the initial treatment with safe, effective, and convenient treatment with ART for PLHIV but who have never taken ARV drugs before—to second-line treatment, to a salvage treatment regimen (i.e., therapy given when the first- and second-line treatments are no longer effective, usually due to toxicity or development of resistance to many of the antiretrovirals). At the current annual cost of HIV treatment of US$134 per patient, the funding needed to achieve universal access to HIV treatment for 1.9 million patients in Nigeria is about US$254.6 million annually. In addition, as PLHIV live longer, requirements for sustainable financing will likely continue to increase, especially if new HIV infections continue to increase.

### Willingness of Patients to Pay for HIV/AIDS Treatment Services

Although there are PLHIV in Nigeria who pay for services at private hospitals, reliable data on the number of such patients paying for HIV treatment services are scant as several private hospitals also benefit from the donor and government-provided ARV services. The willingness of patients to pay could be expressed in the form of paying an insurance premium or paying out-of-pocket for the full or subsidized cost of services. The assessment of willingness to pay can be achieved by the direct method that involves determining the expressed value the user places on the service. It can also be achieved by the indirect method that involves determining tradeoffs between the valued effects of an intervention and the monetary amount involved from which willingness to pay measures are derived.^10^

The most used method in determining willingness to pay for health care is the contingent valuation method. This is a direct, hypothetical, survey-based method used for eliciting a monetary value of a health care service or technology.^11^ The prospective technique determines willingness to pay based on a hypothesized market presented to respondents. We aimed to assess patients' willingness to pay for such services if current funding is no longer available, identify the factors influencing willingness to pay, and explore the feasibility of cost recovery through patient fees in Nigeria.

We aimed to assess patients' willingness to pay for HIV services if current funding is no longer available, identify the factors influencing willingness to pay, and explore the feasibility of cost recovery through patient fees in Nigeria.

## METHODS

### Study Design and Population

The study was a cross-sectional interviewer-administered semistructured survey with PLHIV who were currently receiving free HIV treatment services in 2 states (Lagos and Enugu) and the Federal Capital Territory, Abuja Nigeria. The population for the study consisted of PLHIV who are currently receiving free treatment in Nigeria.

### Sampling and Sample Size

The estimated population of Nigerians who are currently living with HIV is about 2 million, while about 1 million of them are receiving free HIV treatment.^7^ We used purposive sampling method to select 3 clusters (2 states, Lagos and Enugu, and the Federal Capital Territory Abuja (FCT). These 3 states/territories (Lagos, FCT, and Enugu) combined have about 200,000 PLHIV.^8^ Lagos and FCT have a medium-level burden of HIV with prevalence rates of 1.3% and 1.5%, respectively.^8^ Enugu has a high HIV burden state with a prevalence of 2.1%.^8^ We used this sampling frame of PLHIV in these 3 states/territories (Lagos, FCT, and Enugu) to conduct a power analysis at a 95% confidence interval level and a 5% margin of error. This analysis produced a minimum sample size of 399 respondents. Eventually, when we considered a 5% nonresponse rate, a total sample size of 419 was achieved for this study. We applied a formula for calculating sample size from a known population to arrive at the final sample size (Box).

BOX **Formula for Calculating Final Survey Sample Size From a Known Population of People Living With HIV and Receiving Free Treatment Services**

n = NN/1+NN(ee)^2^, where,
n = required sample size
N = population size
e = margin of error (±0.05%)
Therefore, sample size $n=\frac{200,000}{1+200,000(0.05{)}^{2}}$

$n=\frac{200,000}{1+200,000*0.0025}$

$n=\frac{200,000}{2a1+500}$

$n=\frac{200,000}{501}$

Minimum sample size=399

Add 5% nonresponse rate, total sample size=419 respondents

In addition, we chose these settings (Lagos, FCT, and Enugu) because they provided a good mix of populations of different socioeconomic statuses and a mix of urban and rural populations. From a list of all health facilities providing HIV treatment services in each state/territory, a simple random technique without replacement was used to select 5 health facilities from each state to give a total of 15 health facilities. An equal number of health facilities were sampled per state. The health facilities were the comprehensive sites that offer comprehensive HIV treatment services to PLHIV. In each health facility, we used a simple random technique to select the study participants.

On clinic days at each of the 15 facilities, we randomly approached potential participants while waiting for consultation with the doctor or drug refill and adherence counseling at the pharmacy department until the minimum sample size was reached. Our inclusion criteria were respondents who were adults older than age 18 years, living with HIV, receiving free HIV treatment services in Nigeria, and who provided informed consent to participate. Only respondents who had access to HIV treatment for more than a year and have not missed appointments were included. Respondents who did not meet the above criteria were excluded.

### Data Collection

An interviewer-administered semistructured questionnaire was used to elicit respondents' willingness to pay for HIV treatment services. The survey instrument was adapted from previous studies on willingness to pay for health commodities or services conducted in Nigeria and several other African countries.^12^ We adapted the survey instrument to meet the purpose of this study by incorporating bidding amounts that align with the current estimated average cost of obtaining HIV treatment services in Nigeria. Data were collected on the socioeconomic status of respondents, the existence of comorbidity, history of payment for existing comorbidity, history of payment for HIV treatment services, and types of treatment services paid for, among other information. The survey instrument was administered between January 29 and March 22, 2020. A total of 400 eligible PLHIV from the 15 health care facilities provided informed consent and participated in the survey.

### Bidding Game Iteration Method

The bidding method was used to obtain information on the willingness of respondents to pay for HIV services and the maximum amount respondents were willing to pay. The treatment services include clinical services, consultations, laboratory investigations, and supplies at the estimated cost of $140 per year. During the bidding, we asked the respondents if they were willing to pay the current estimated price of 5,000 naira (US$12.5) per month to access treatment. Respondents who answered “Yes” to the question were asked if they were willing to pay for treatment if the price increased to 6,000 naira (US$15). For each “Yes” response received, respondents were asked about their willingness to pay if the price increased further until a “No” response was received. However, respondents who answered “No” to any price bid were asked if they would be willing to pay for treatment services if the price was lower. The maximum amount a respondent was willing to pay was determined from the highest price point at which the respondents provided a “Yes” answer to a bid that was followed by a “No” response.

### Data Management and Analysis

Descriptive analysis was conducted using SPSS version 23, and results were summarized by the number, percentages, mean, median, standard deviation, and range. In addition, we conducted bivariate analysis by testing the association between 17 key socioeconomic and patient-factor variables and willingness to pay (primary dependent variable) versus the maximum amount respondents were willing to pay (secondary dependent variable).

Bivariate analysis was conducted to determine the association between willingness to pay (dependent variable) and independent variables. Chi-square (X^2^) and *P*-values were recorded for each variable. Also, multivariate logistics regression analysis was conducted to determine the degree of association between willingness to pay and independent variables. The results were interpreted in terms of odds ratios and *P*-value at a 95% confidence interval (CI). A demand curve was developed from the collected data and the price elasticity of the amounts that patients were willing to pay per month relative to the estimated current cost of providing HIV treatment services per patient was determined as well as the patient coverage and potential cost recovery at each price points were determined.

### Ethics Approval and Consent to Participate

The study protocol was reviewed and approved by the National Health Research Ethics Committee (NHREC) of the Federal Ministry of Health in Nigeria (NHREC/01/01/2007-23/12/2019) and the Institutional Review Board of the University of North Carolina at Chapel Hill (Institutional Review Board Notice 18-2723). We sought consent from each participant and only included those who gave consent.

## RESULTS

### Sociodemographic Characteristics of Respondents

Of the 419 respondents that we approached, 400 respondents completed the questionnaire, yielding a response rate of 96%. This formed the basis for analysis. The mean and median ages of respondents were 36.1 and 34 years, respectively. About 53% of the respondents were females while 47% were males. Analysis of the marital status of respondents indicated that about 32% of the respondents were single, 40% married, 7.2% not married but cohabiting with a partner. About 90% of respondents had at least a primary education while only 10% never attended any formal education. About 79% had a regular income either through paid employment or through informal employment or business, whereas, about 10% of respondents were students and 11% were not employed. The median monthly income of respondents was 28,000 naira (US$70). Among those who were employed, 32% earned less than the national monthly minimum wage, while 44% earned above the minimum wage. More than half (58%) of survey respondents had been on HIV treatment for more than 3 years. About 86% of respondents were on first-line ART, while 14% were on second-line treatment (Table 1).

**TABLE 1.** Demographic Characteristics of Respondents to Survey of People Living With HIV Currently Receiving Free HIV Treatment Services in Nigeria (N=400)

	No. (%)
Age, mean (±SD); median (IQR)	36.1(±10.1); 34 (18–74)
Age group	
18–28 years	91 (22.75)
29–39 years	174 (43.00)
40–50 years	100 (25.00)
51–61 years	27 (6.75)
Older than 62 years	8 (2.00)
Sex	
Female	211 (52.72)
Male	189 (47.25)
Marital status	
Cohabiting	32 (8.00)
Divorced/separated	39 (9.72)
Married	161 (40.25)
Single	126 (31.50)
Widowed	42 (10.50)
Level of education completed	
No education	38 (10.00)
Primary	81 (21.00)
Secondary	153 (38.25)
Postsecondary	117 (29.00)
Others	48 (12.00)
Employment status	
Salaried	121 (30.25)
Self-employed	196 (49.00)
Students	38 (9.50)
Unemployed	45 (11.25)
Monthly income	
No income	76 (19.00)
Less than 10,000 naira (<US$25)	10 (2.50)
10,000–20,000 naira (US$25–50)	56 (14.00)
20,000–30,000 naira (US$50–$75)	71 (17.75)
30,000–40,000 naira (US$75–$100)	93 (23.25)
50,000–100,000 naira (US$125–$250)	67 (16.75)
Above 100,000 naira (>US$250)	16 (4.00)
Would not disclose	11 (2.75)
Frequency of visit to the clinic	
Once a month	86 (21.50)
More than once a month	5 (1.25)
Once every 2 months	75 (18.75)
Once every 3 months	234 (58.50)
Duration on treatment	
Less than 1 year	36 (9.00)
Between 1–3 years	132 (33.00)
More than 3 years	232 (58.00)
Type of HIV treatment being received	
First-line treatment	344 (86.00)
Second-line treatment	56 (14.00)

### Willingness to Pay for HIV Treatment Services

Most 366 (92%) respondents indicated that they would be willing to pay for HIV treatment services if services were no longer available for free. In addition, they were asked what decision they would make about their HIV treatment if payment was required. All those who responded “Yes” to the initial question indicated that “they would try to look for ways to pay for treatment.” Those who responded “No” reported that they would discontinue their HIV treatment if such a situation arose. Despite the high willingness to pay for HIV treatment, 68% of the respondents believed it was not acceptable for patients to pay for treatment as it was perceived to be the responsibility of the government to provide free HIV treatment for all those who need the treatment. The median amount respondents would be willing to pay was 2,000 naira (US$5) per month with only about 18% willing to pay the monthly cost of treatment of 5,000 naira (US$12.5).

If HIV treatment services were no longer provided for free, 92% of survey respondents indicated they would be willing to pay for services.

### Willingness to Pay for HIV Treatment With Changes in Income

**FIGURE 1:**
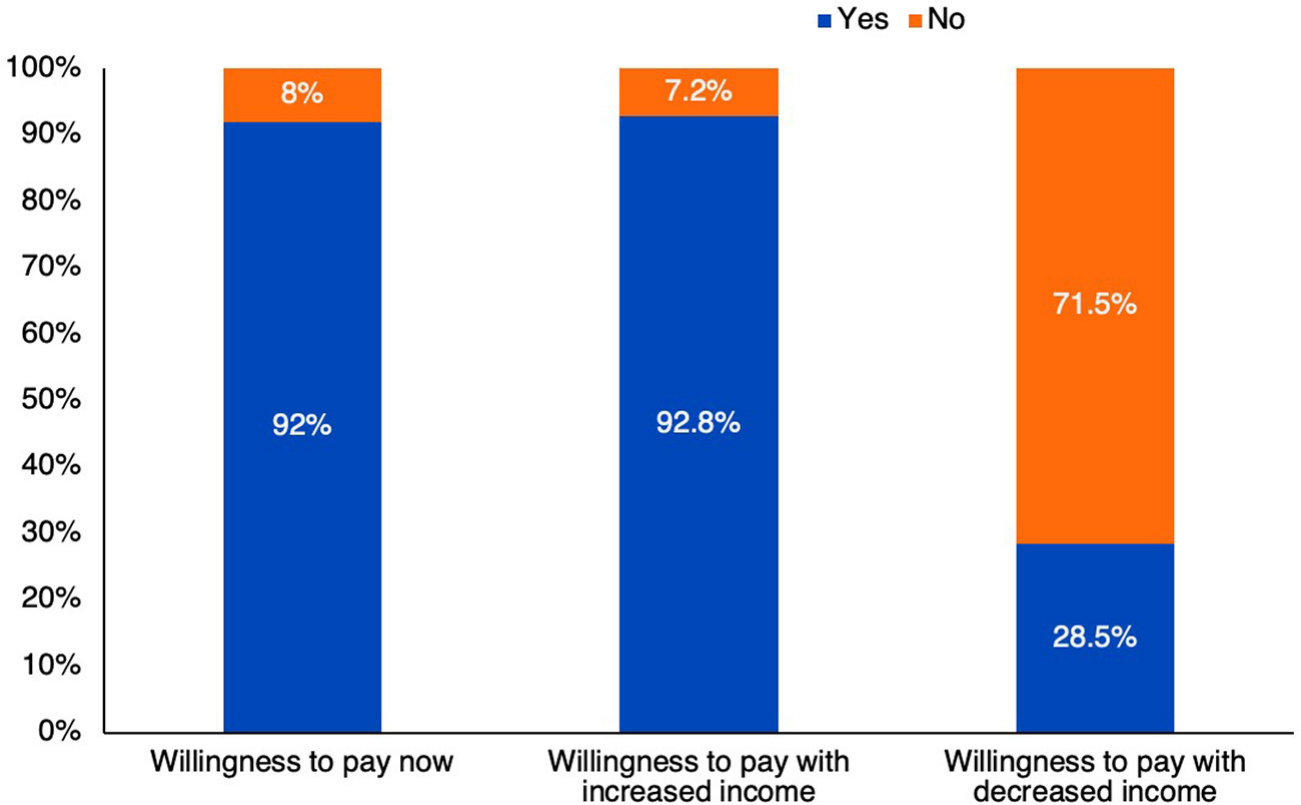
Respondents' Willingness to Pay for HIV Treatment Services With Changes in Income, Nigeria

Irrespective of the response on willingness to pay, respondents were asked if their willingness to pay would change with any increase or decrease in their income. Of 400 respondents, 371 (92.8%) were willing to pay if income increased. Also, about 28.5% of all respondents would be willing to pay if their income decreased while 71.5% would not be willing to pay if income decreased (Figure 1).

### Maximum Amount Respondents Were Willing to Pay

**FIGURE 2:**
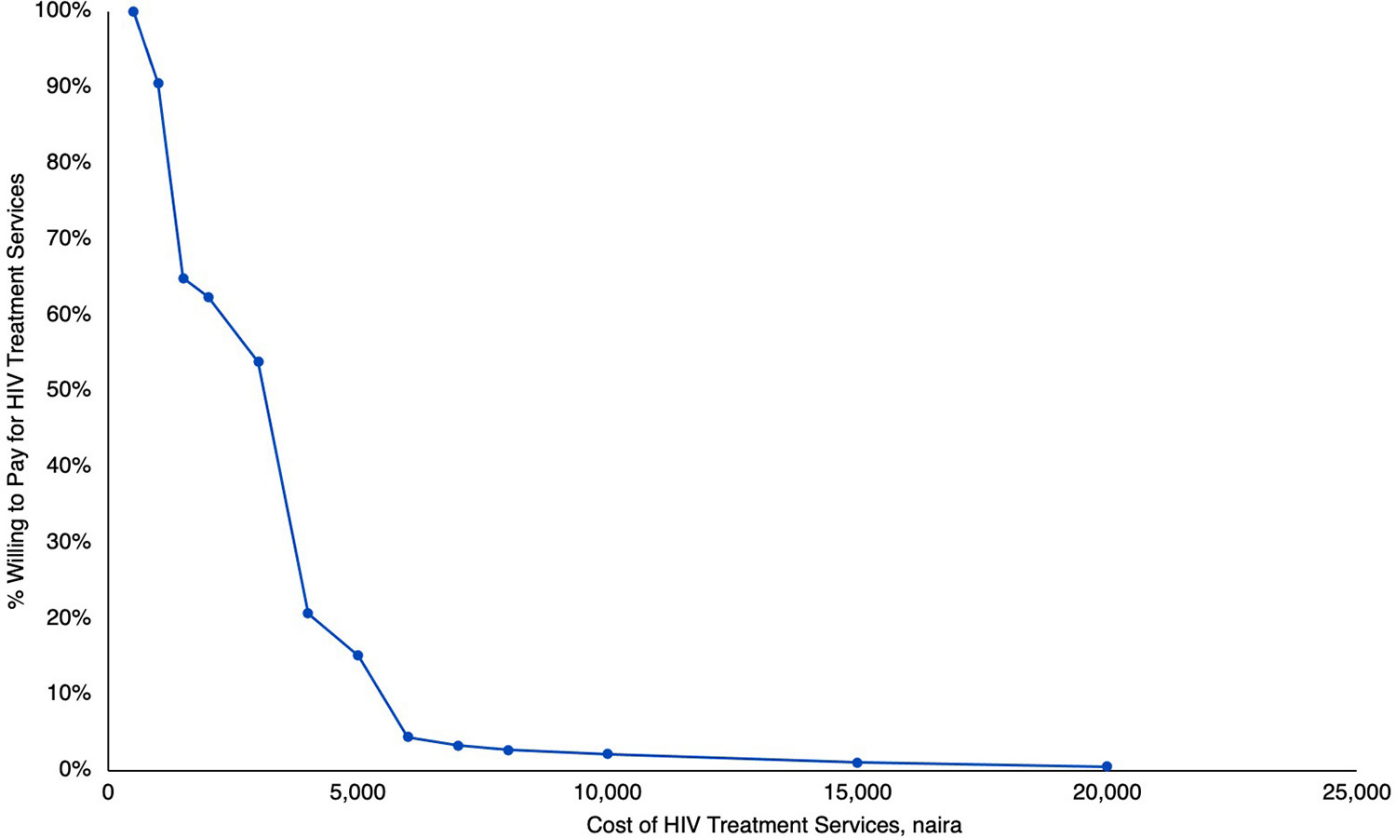
Percentage of Respondents' Willingness to Pay at Different Price Points of HIV Treatment Services (Demand Curve) in Nigeria

Figure 2 shows the variation of willingness to pay for HIV treatment services at different treatment price points. The maximum amount respondents were willing to pay ranged from 500 naira to 20,000 naira (US$1.25–$50) with the median amount of 2,000 naira (US$4). Only 18% of respondents indicated a willingness to pay the current estimated cost of 5,000 naira (US$12.5) per month.

### Preferred Type of Health Facilities and Sources of Funds for HIV Treatment Services

About 90% of the respondents preferred a public health care facility while about 10% preferred a private health care facility. Respondents preferred a public facility due to the availability of comprehensive treatment services (drug refill, consultation, and laboratory services) at such locations while those who prefer private health care facilities cited shorter waiting times and a higher level of confidentiality at such centers for their care.

Respondents who were willing to pay for treatment were asked what the source(s) of the funds for payment would be. More than two-thirds (71%) of patients who expressed willingness to pay for treatment reported that the funds would come from a combination of personal income and support from family and friends. Further, 20% indicated that the funds would come solely from personal income while 9% reported the funds will come from support from family and friends. However, 84% of respondents indicated they were sure that they could access financial support from family and friends to pay for HIV treatment services if such support was required (Table 2).

**TABLE 2.** Survey Respondents' Willingness to Pay for HIV Treatment Services in Nigeria (N=400)

Variables	No. (%)
Willing to pay for treatment if free treatment is stopped	366 (91.50)
Willingness to pay with increasing income	371 (92.75)
Willingness to pay with decreasing income	114 (28.50)
Sources of payment for treatment	
Family/friends	34 (9.28)
Personal income	80 (21.86)
Personal income with family/friends	252 (68.85)
Type of health facility preferred for paid services	
Government hospital	327 (89.83)
Private hospital	37 (10.08)
Preferred mode of payment	
Annual payment to the hospital	37 (10.11)
Payment on the day you come to the hospital	272 (74.32)
Payment through health insurance premium	57 (15.57)

### Preferred Mode and History of Payment for HIV Treatment Services

Respondents who were willing to pay for HIV treatment services were asked their preferred mode of payment and 75% would prefer to pay out of pocket on the day of the clinic visit as this would limit the burden of payment. In addition, 11% would prefer to make annual payments to the clinic where services are being provided while 14% would prefer payment through a health insurance scheme (HIS). Over half (58%) reported they paid for treatment services at least once since they were diagnosed with HIV while 42% have never made any form of payment. Among those who ever paid for HIV treatment (N=232), 83% made the payment in a publicly owned health facility while 17% paid in a privately owned health facility. About 3% of respondents paid for registration for consultation, 56% paid for laboratory services only, while 41% paid for multiple services including laboratory tests, registration for consultation, and drug refill. The median amount reported by respondents was 5,000 naira (US$12.5) while the reported amount ranged from 1,000 naira to 15,000 naira (US$2.5–US$37.5) per clinic visit. About 7% of the respondents paid up to N1000 (US$2.5), 27% paid up to 2,000 naira (US$5), 38% paid up to 4,000 naira (US$10), 18% paid up to 6,000 naira (US$15) while 10% paid more than 6,000 naira (US$15) for previous treatment services.

### Bivariate Analysis of Independent Variables With Willingness to Pay for HIV Treatment Services

To determine the statistical association between independent variables and the dependent variable (willingness to pay for treatment), we conducted bivariate analysis and documented Chi-square values and *P*-values (Table 3).

**TABLE 3.** Bivariate Analysis of Survey Respondents' Willingness to Pay for HIV Treatment Services in Nigeria (N=400)

Variable	Respondent Willing to Pay, No. (%)	X^2^	P Value
Sex			
Male	175 (92.6)		.51
Female	191 (90.5)	0.45	
Age category			
Older than 40 years	242 (91.3)		.95
Younger than 40 years	124 (91.9)	2.50	
Marital category			
Has a partner	182 (94.3)		.07
Has no partner	184 (88.9)	3.38	
Educational category			
Below secondary school	107 (89.9)		.95
Above secondary school	259 (92.2)	0.00	
Employment category			
Employed	301(94.3)		.001^a^
Unemployed	65 (75.9)	22.6	
Monthly income			
>Minimum wage	184 (98.4)		.001^a^
<Minimum wage	182 (85.4)	14.9	
Change in income			
Decrease in income	114 (28.5)		.001^a^
Increase in income	371 (92.8)	7.79	
Availability of support			
No support	43 (62.3)		.001^a^
Family and friend support	318 (96.1)	49.17	
Sources of money to be used to pay for HIV treatment services			
Personal income plus support	275 (96.8)		
Personal income alone	67 (91.8)	3.42	.06
Perception of the monthly cost of HIV treatment services			
Cost is low	170 (92.9)		
Cost is high	196 (90.3)	0.66	.42
History of previous payment for HIV treatment services			
No	149 (89.8)		
Yes	217 (92.7)	1.24	.27
Awareness of benefits of treatment			
No	5 (71.4)		
Yes	361 (91.9)	11.36	.001^a^
Duration the respondents have been on HIV treatment			
Less than 3 years	150 (89.8)		
More than 3 years	216 (92.7)	1.17	.28
Preferred location to receive HIV treatment services			
Public hospital	35 (95)		
Government hospital	320 (98)	2.35	.33
Existence of comorbidity with HIV infection			
No comorbidity	345 (99.7)		
Yes comorbidity	21 (38.9)	0.91	.50
Frequency of clinic visits by respondents			
More than once a quarter	141 (84.9)		
Once in a quarter	225 (96.2)	3.06	.80
Awareness of the benefits of adherence to HIV treatment services			
No	5 (71.4)		
Yes	361 (91.9)	2.22	.14

a Statistically significant *P* values of .05. Only respondents who expressed willingness to pay were asked this question (n=366).

#### Availability of Financial Support From Friends and Family

The willingness to pay for HIV treatment services was higher if the respondents receive support from family and friends (X2=49.17; *P*<.001*). Respondents who had access to financial support from family and friends had a higher willingness to pay for HIV treatment services compared to those who lacked such support (Table 3).

#### Awareness of Benefits of Early HIV Treatment Initiation

Respondents who were aware of the benefits of the early HIV treatment initiation were more likely to pay for HIV treatment services (X^2^=11.36; *P*<.001) (Table 3).

#### Change in Monthly Income

Changes in income (increase) were associated with the respondent's willingness to pay for HIV treatment services. The more the respondents' income increased, the more they were willing to pay for HIV treatment services (X^2^= 7.79; *P*<.001).

#### Monthly Income Level

The Chi-square test was statistically significant for the bivariate analysis of monthly income and willingness to pay (X^2^=14.9; *P*<.001). The finding indicates that the respondents may not pay for HIV treatment services if their income was below the national minimum wage. Respondents who earned monthly income below the national minimum wage of 30,000 naira (US$75) per month were less likely to pay for HIV treatment services. The willingness to pay increased with increasing income above the minimum wage of respondents.

#### Employment Category

Although the employment category was also statistically significant (X^2^=22.6; *P*<.001), the chances of the respondents paying for HIV treatment services were lower if they were unemployed compared to those employed.

### Bivariate Analysis of Independent Variables With Maximum Amount Respondents Were Willing to Pay for HIV Treatment Services

The current cost of providing HIV treatment services in Nigeria was estimated by NACA in 2018 to be about 5,000 naira (US$12.5) per patient per month. Therefore, data collected on the maximum amount respondents were willing to pay were recoded into a dichotomous variable of below 5,000 naira (<US$12.5) and 5,000 naira and above (≥US$12.5). We describe the findings from the bivariate analysis of how this dependent variable is predicted by the demographic and socioeconomic factors of the respondents (Table 4).

**TABLE 4.** Output of Bivariate Analysis of Independent Variables With Maximum Amount Respondents Are Willing to Pay for HIV Treatment Services in Nigeria (n=400; 95% CI)

Variable	Max Amount Willing to Pay, No. (%)>N5,000 (US$12.5)	X^2^	P Value
Sex			
Male	27 (14.3)		
Female	20 (9.5)	1.38	.24
Age			
Older than 40 years	20 (14.8)		
Younger than 40 years	27 (10.2)	0.37	.54
Marital category			
Has a partner	31 (16.1)		
Has no partner	16 (7.7)	1.06	.15
Education completed			
Below secondary school	23 (19.3)		
Secondary school and above	24 (8.5)	6.33	.012^a^
Employment category			
Employed	39 (12.3)		
Not employed	8 (9.6)	2.0	.16
Monthly income			
>Minimum wage	36 (19.3)		
<Minimum wage	11 (5.2)	3.45	.001^a^
Change in income			
Decrease in income	137 (38)		
Increase in income	116 (32)	0.002	.98
Availability of support			
Family and friend support	37 (11.1)		
No support	10 (14.7)	23.67	.001^a^
Sources of money to be used to pay for HIV treatment services			
Personal income alone	39 (10.9)		
Personal income plus support	8 (18.6)	2.93	.06
Perception of the monthly cost of HIV treatment services			
Cost is low	41 (11.7)		
Cost is high	7 (14)	1.13	.42
History of previous payment for HIV treatment services			
No	26 (15.7)		
Yes	21 (9)	0.22	.27
Awareness of benefits of treatment			
No	0 (0)		
Yes	47 (12)	0.01	.99
Duration the respondents have been on HIV treatment			
Less than 3 years	11 (6.6)		
More than 3 years	36 (15.5)	1.02	.33
Preferred location to receive HIV treatment services			
Public hospital	1 (2.7)		
Government hospital	46 (14.1)	1.05	.33
Existence of co-morbidity with HIV infection			
No	40 (10.6)		
Yes	7 (31.8)	0.91	.50
Frequency of clinic visits by respondents			
More than once a quarter	11 (6.6)		
Once in a quarter	36 (15.4)	0.59	.80
Awareness of the benefits of adherence to HIV treatment services			
No	0 (0)		
Yes	47 (12)	3.58	.14

a Statistically significant *P* value of .05.

Three of the 17 independent variables investigated were observed to have a statistically significant association with the maximum amount that respondents were willing to pay for HIV treatment services. The *P*-values for the variables were less than .001, thereby rejecting the null hypothesis that willingness to pay for HIV treatment services was independent of these factors.

#### Availability of Financial Support From Friends and Family

We found an association between access to financial support from family and friends and the maximum amount the respondents were willing to pay. The respondents who had access to financial support from family and friends were willing to pay an amount above 5,000 naira (US$12.5) per month for HIV treatment services compared to those who did not have such support (X^2^=23.67; *P*<.001).

#### Level of Education Completed

Bivariate analysis of the association between the maximum amount respondents were willing to pay and the maximum education completed (secondary school and above) was statistically significant (X^2^=6.33; *P*<.001). The respondents who completed secondary education or higher indicated a higher willingness to pay an amount above the current price of 5,000 naira (US$12.5).

#### Monthly Income

The monthly income of respondents was statistically significant when associated with the maximum amount respondents were willing to pay (X^2^=3.45; *P*<.001). Specifically, respondents who earned lower than the minimum wage were less willing to pay a maximum amount greater than 5,000 naira (US$12.5) compared to those who earned above the minimum wage.

### Multivariate Logistics Regression of Willingness to Pay for HIV Treatment Services and Independent Variables

We conducted logistics regression to assess the magnitude of the association between independent variables and the dependent variable (willingness to pay for treatment). The results were expressed in terms of odds ratios (OR) at 95% CI. Looking at the individual predictor factors, employment, changes in income, and availability of financial support from families and friends were statistically significant with *P*-values of less than .001 (Table 5).

**TABLE 5.** Multivariate Regression Analysis of Respondents' Willingness to Pay for HIV Treatment Services in Nigeria and Independent Variables^a^ (N=400)

Model	Odds Ratio (95% Confidence Interval)	P Value
Age category		
Older than 40 years		
Younger than 40 years	0.934 (−0.091, 0.032)	.351
Marital category		
Has a partner		
Has no partner	0.130 (−0.060, 0.052)	.897
Educational category		
Below secondary school		
Secondary school and above	0.355 (−0.064, 0.044)	.723
Employment category		
Employed		
Not employed	0.190 (0.015, 0.202)	.023^b^
Monthly income		
>Minimum wage		
<Minimum wage	2.389 (−0.046, 0.069)	.697
Sex		
Male		
Female	0.288 (−0.089, 0.019)	.199
Level of treatment		
Public hospital		
Government hospital	0.770 (−0.086, 0.038)	.442
Duration on treatment		
Less than 3 years		
More than 3 years	1.274 (−0.015, 0.070)	.204
Co-morbidity with HIV		
No		
Yes	0.119 (−0.110, 0.124)	.905
Change in income		
Decrease in income		
Increase in Income	2.015 (0.003, 0.229)	.045^b^
Availability of financial support		
Family and friend support		
No support	14.209 (0.151, 0.285)	.00^b^
Sources of money to be used to pay for HIV treatment services		
Personal income alone		
Personal income plus support	0.548 (1.523, 20.24)	.802
Perception of the monthly cost of HIV treatment services		
Cost is low		
Cost is high	1.493 (0.398, 2.273)	.892
Awareness of consequences of nonadherence to HIV treatment		
No		
Yes	1.856 (2.753, 10.274)	.136
Awareness of starting treatment as soon as a diagnosis is completed		
No		
Yes	0.998 (3.02, 65.507)	.075
History of previous payment for HIV treatment services		
No		
Yes	0.576 (0.443, 32.740)	.144
Frequency of clinic visits		
More than once a quarter		
Once in a quarter	2.457 (0.122, 0.523)	.784

a Prob >F=0.000.

b Statistically significant *P* value of .05.

#### Employment Status

The likelihood of paying for treatment services was higher among the patients who were employed at the time of the study (OR=0.190; *P*<.001; 95% CI=0.015, 0.202). This implies that if patients are employed and have a source of primary income, they are more likely to be willing to pay for treatment services.

#### Change in Income

Logistics regression analysis predicted that changes in income were largely associated with willingness to pay for treatment services (OR=2.015; *P*<.001; 95% CI=0.003, 0.229). An increase in income was associated with a higher willingness to pay for HIV treatment services. Therefore, the more the income decreases, the less likely patients would be willing to pay for HIV treatment services.

### Availability of Financial Support From Families and Friends

Availability of financial support was 14.2 times associated with willingness to pay for HV treatment services. The more patients had support from family and friends, the more likely they were willing to pay for treatment services (OR=14.209; *P*<.001; 95% CI=0.151, 0.285) Table 5.

### Multivariate Logistics Regression Analysis of Maximum Amount Respondents Were Willing to Pay and Independent Variables

We conducted a multivariate logistics regression analysis of the maximum amount the respondents were willing to pay for HIV treatment and all 17 independent variables (Table 6).

**TABLE 6.** Multivariate Regression Analysis of Maximum Amount Respondents Were Willing to Pay for HIV Treatment Services in Nigeria with Independent Variables^a^ (N=400)

Model	Odds Ratio (95% Confidence Interval)	P Value
Age category		
Older than 40 years		
Younger than 40 years	1.003 (−581.887, 117.994)	.19
Marital category		
Has a partner		
Has no partner	0.699 (−204.520, 429.975)	.49
Educational category		
Below secondary school		
Secondary school and above	0.797 (−181.817, 429.592)	.43
Employment category		
Employed		
Not employed	1.168 (−215.269, 844.746)	.24
Awareness of early start of treatment		
No		
Yes	0.646 (−254.997, 504.569)	.52
Sex		
Male		
Female	0.948 (−452.638, 158.094)	.34
Monthly income		
>Minimum wage		
<Minimum wage	2.476 (84.698, 737.233)	.014^a^
Change in income		
Decrease in income		
Increase in income	4.332 (1222.577, 505.391)	.00^a^
Comorbidity with HIV		
No		
Yes	0.821 (−385.006, 937.531)	.41
Availability of financial support		
Family and friend support		
No support	1.092 (−567.603, −87.090)	.008^a^
Level of treatment		
Public hospital		
Government hospital	0.383 (−418.816, 282.278)	.70
Sources of funds		
Personal income alone		
Personal income plus support	1.072 (0.053, 3.071)	.39
Perception of cost of HIV treatment services		
Cost is low		
Cost is high	1.18 (0.034, 23.345)	.99
Awareness of consequences of nonadherence to treatment		
No		
Yes	1.175 (−432.34, 123.345)	.67
Frequency of clinic visits		
More than once a quarter		
Once in a quarter	0.881 (0.072, 10.804)	.92
Duration on treatment		
Less than 3 years		
More than 3 years	0.457 (1.325, 7.824)	.73

a Prob >F=0.000

Availability of financial support from family and friends, monthly income, and change in monthly income was statistically significant at *P*-value less than .001 while other independent variables considered were not statistically significant. Hence, improvement or upward change in socioeconomic factors was important in influencing the maximum amount respondents were willing to pay for their treatment if the free treatment was stopped (Table 6).

#### Monthly Income

Monthly income level was associated with the maximum amount respondents were willing to pay for HIV treatment services (OR=2.476; *P*<.001; 95% CI=84.698, 737.233). When the income level was above the minimum wage, the likelihood of paying a maximum amount above 5,000 naira (US$12.5) (cost of treatment) increased compared to those who earned below the minimum wage.

#### Change in Monthly Income

Change in income (specifically, increase in income) had a positive association with willingness to pay (OR=4.332; *P*<.001; 95% CI=1222.577, 2505.391). When income increased, willingness to pay for treatment also increased. When income increased, willingness to pay increased. A higher income increased the likelihood of being willing to pay a maximum amount greater than 5,000 naira (US$12.5).

#### Availability of Financial Support

Availability of financial support was strongly associated with willingness to pay for HIV treatment services. Respondents who had access to financial support from friends and family had a higher willingness to pay for HIV treatment services. Also, the availability of financial support from family and friends was associated with the maximum amount the respondents were willing to pay for HIV treatment services (OR=1.092; *P*<.001; 95% CI=−567.603, −87.090). Access to financial support increased the likelihood of paying an amount greater than 5,000 naira (US$12.5) compared to those who did not have access to such support (Table 6).

### Potential Cost Recovery, Elasticity, and Patient Coverage Through Patients' Willingness to Pay for HIV Treatment Services

Evidence suggests that the marginal cost of HIV treatment per person per month in Nigeria is about 5,000 naira (US$12.5).^8^ This value was used as the reference point for the consideration of the price elasticity, patient coverage, and potential cost recovery for HIV treatment in Nigeria. Relative to the current monthly cost of treatment, the price point of 3,000 naira had the highest price elasticity with an estimated 54% of patient coverage and 29% cost recovery. However, the price point of 4,000 naira had the lowest price elasticity of 0.63 with 21% patient coverage and an estimated 15% cost recovery. At the price point of 500 naira (US$1.25), the price elasticity is 0.85 with about 91% of the patient load covered, but only 9% cost recovery would be achieved. Table 7 presents the relevant results from the different price points on the demand curve.

**TABLE 7.** Elasticity of Price for HIV Treatment in Nigeria, Patient Coverage, and Potential Cost Recovery

Willingness to Pay Price Points	Elasticity @ Naira 5,000	Patient Coverage, %	Potential Cost Recovery, %
Naira 500 (US$1.25)	0.85	91	9
Naira 1,000 (US$2.50)	0.85	65	12
Naira 2,000 (US$5.00)	1.29	62	23
Naira 3,000 (US$7.50)	2.0	54	29
Naira 4,000 (US$10.00)	0.63	21	15
Naira 5,000 (US$12.50)		18	16

## DISCUSSION

We aimed to assess the willingness of patients receiving free HIV treatment to pay for services and the factors that influence their decisions. These findings can help estimate potential cost recovery to improve funding sustainability for HIV treatment in Nigeria. To our knowledge, this is the first application of the findings from a willingness to pay for HIV treatment in Nigeria to estimate potential cost recovery to improve funding sustainability for HIV treatment programs in Nigeria.

Overall, 92% of patients expressed they would be willing to pay for HIV treatment services if free services were no longer available. The observed high proportion of patients is surprising for a service that has been provided free to patients in Nigeria for more than a decade. However, the wide variation in the amount that patients were willing to pay did not come as a surprise. Considering that almost all the patients reported that they were aware of the consequences of interrupting treatment for HIV/AIDS due either to nonadherence to ART or lack of clinical monitoring through laboratory investigations, such awareness could have influenced their willingness to contribute to funding their treatment. Adherence to HIV treatment has been identified to be critical in optimizing patients' responses to therapy.^13^ Although long-term adherence to ART is perceived as a compromise between conflicting demands of everyday work,^14^ almost all patients appreciated and valued the importance of adherence to their treatment and thus, their willingness to pay. This could also be because most of the respondents in our study have been on HIV treatment for 1–3 years. The proportion of patients who were willing to pay for HIV treatment services obtained from this study is one of the highest ever recorded in any study conducted in Africa. This could be due to a higher literacy level of respondents in our study which enabled them to appreciate the importance of adherence to treatment or the fact that the cost of accessing treatment for HIV has continued to reduce over the years, thus becoming more affordable. Our study used the bidding approach to evaluate the willingness of patients to pay for HIV treatment services in Nigeria. Comparatively, a study conducted in southeast Nigeria found that about 67% of patients were willing to pay for HIV treatment services, which is different from our findings.^15^ However, our findings were similar to another study which indicated that about 91% of patients were willing to pay for HIV treatment services.^16^

The proportion of patients who were willing to pay for HIV treatment services obtained from this study is one of the highest ever recorded in any study conducted in Africa.

Despite the high proportion of patients who expressed willingness to pay for HIV treatment services in Nigeria, the amount expressed by patients varied significantly. Most patients indicated that HIV treatment should be provided free of charge by the government and if such provision could not be made by the government of Nigeria, this amounts to a failure of governance. A study suggested that patients would be willing to pay a higher amount than the actual cost of the service if they valued such health service.^17^ This may not be true if patients do not possess the financial capacity to pay for service even if the health service is perceived to be important. Despite most patients rating the quality of HIV treatment services to be very good, the maximum amount patients were willing to pay decreased with the increasing cost of the service during the bidding game iteration process. Only about 18% of patients were willing to pay the current cost of monthly ARV services at 5,000 naira (US$12.5).

With more than 1.2 million patients currently on HIV treatment, the expression of willingness to pay the current cost of treatment by a fraction (18%) of the patients will potentially increase funding for HIV treatment services by a minimum of 14 billion naira (US$36 million) annually. In addition, this is an indication to policy makers and HIV implementers in Nigeria that patients are willing to contribute to their treatment, and this can serve as cost recovery to promote the sustainability of HIV treatment services in Nigeria. We estimated that the price point of 3,000 naira (US$7.5) per month provides the greatest price elasticity with the potential for recovery of about 29% of the total cost of providing treatment. Although only about 54% of patients were willing to pay this price, if the price is reduced to 2,000 naira (US$10) per month, more patients (62%) will be willing to pay for treatment. However, this amount will cover only 23% of the total cost of treating all patients. Further reducing the monthly cost of treatment will enhance affordability and enable a larger number of patients to contribute to domestic funding for HIV treatment in Nigeria.

Despite such potential for additional domestic funding, an expression of willingness to pay for a health service does not necessarily translate to a capacity to pay. Some of the patients may not have thought through the long-term implications of paying to access health services for a chronic health condition. Even if expressed willingness to pay for the services translates to a capacity to pay, some patients may default in payments due to competing financial demands. Therefore, such a funding source may be unreliable due to the inconsistency of payment by patients. A way forward to improve the reliability of this source of funding would be to have payments paid via insurance premium which can help pool resources from both healthy and sick populations with different levels of income rather than out-of-pocket payment by the patients. Such a payment method will likely protect patients from financial hardship and ruin due to ill health.^18^

However, more than two-thirds of the patients who expressed willingness to pay for treatment preferred payment out-of-pocket at the point of care rather than through a health insurance premium. Our finding aligns with the finding that less than 5% of Nigerians are currently enrolled in health insurance schemes in Nigeria.^19^ The reason ascribed by the patients for this preference is their perception of a lack of transparency in the health insurance program in Nigeria as well as challenges of information asymmetry and poor quality of services experienced by those who currently have health insurance. These failures of the health insurance market, in addition to “cream skimming” by health insurance providers due to their reluctance to cover preexisting health conditions such as HIV/AIDS, limit PLHIV in purchasing health insurance in Nigeria.^20^ This is why out-of-pocket expenditure as a percentage of total health care costs in Nigeria has remained above 70% for the last decade.^21^ This preference for out-of-pocket payment could also be because some patients feel more comfortable with monthly payments of a lower amount to access HIV treatment services rather than a higher annual payment as the former limits the financial burden. This opinion was buttressed by our findings that about 20% of patients who expressed willingness to pay for treatment would source the funds solely from their personal income while about 70% would source funds from a combination of personal income and support from family and friends. This finding could also be an indication of the low socioeconomic status of most patients receiving HIV treatment services in Nigeria.

Although a significant number of patients expressed willingness to pay for HIV treatment, most offered to pay an amount that was far less than the current cost of services. Their reason was they perceived the provision of free HIV treatment services as the government of Nigeria's social responsibility, and patients should never need to pay for HIV treatment. This perception is often supported by the argument that failure to provide free HIV treatment services will negatively affect patients, caregivers, and the economy, so the services must be free. Without free services, patients and caregivers will experience a loss of output and income and incur costs of frequent hospital visits, inpatient care, and death and burial.^22^ These scholars further argue that living with HIV comes with stigma and discrimination, and PLHIV have higher costs, especially for transportation to get to health care centers. Unlike patients who suffer from other chronic health conditions, most PLHIV seek care at health facilities that are far away from where they live or work to avoid stigma and discrimination.^22^

### Factors That Influence the Willingness of PLHIV to Pay for HIV Treatment

Our bivariate analysis indicated that 5 factors were strongly associated with the willingness of patients to pay for HIV treatment services. The availability of financial support from family and friends had the strongest association with willingness to pay, followed by the level of awareness of the benefits of early initiation of treatment by patients, increase in income, current income level, and the patient's employment status. These findings did not come as a surprise considering that 4 of the 5 identified factors are related to patients' socioeconomic status. These findings are consistent with those from previous studies that found that socioeconomic factors are associated with the willingness of patients to pay for health care services.^15^^,^^23^ Health care is considered a social good and the willingness to pay for such social goods is influenced largely by the financial capacity of patients. Our study yielded a higher percentage of respondents who earn monthly income (79%) compared to prior studies that had 75% and 62%, respectively.^15^^,^^22^ However, there is variation among the specific socioeconomic factors that were associated with willingness to pay for HIV treatment from our study and previous studies from the literature. While several studies have previously revealed that willingness to pay for health care services was associated with the level of education, marital status, distance from home to the clinic, and area where patients live, our findings did not identify any statistically significant association with these factors.^24^^–^^27^ Rather, our study found patients who had education above secondary school level were more likely to be willing to pay a higher amount for HIV treatment compared to those who had education below secondary school level, but this was not statistically significant. A possible reason for this observation could be the ability of individuals who had post-secondary school education to attract higher-paying jobs. This could also be due to these individuals' higher literacy levels that contribute to a greater understanding of the funding challenges of the HIV program in Nigeria.

The availability of financial support from family and friends had the strongest association with willingness to pay.

Family and friends play a significant role in providing psychological, emotional, and financial support to patients undergoing long-term treatment.^22^ This is a common phenomenon in Nigeria and most African countries where people live a communal life and rely on family and friends for support. This support could be actual payment for health care services or economic empowerment in terms of providing job opportunities. In all, such support helps to improve patients' economic status temporarily or permanently and probably leads to increased willingness to pay for HIV treatment as observed from the strong association between the availability of support from friends and family and willingness to pay for HIV treatment in our study.

The role of employment status, level of income, changes in income, and availability of financial support in the willingness and capacity of an individual to pay for health care services cannot be overemphasized. This is because there are competing needs for limited financial resources, and an individual would have to sacrifice payment for these competing needs to pay for HIV treatment. Although about 80% of patients in our study indicated having a source of income, the median income was about 28,000 naira (US$70) per month. This value is below the national minimum wage in Nigeria and far less than the average monthly income of a working Nigerian which is about 85,000 naira (US$213) (salary explorer, 2020). This low level of income among patients limits the willingness of patients to pay about 5,000 naira (US$12.5) per month as this will take up more than 15% of an average patient's monthly income. Therefore, employment status and monthly income play a critical role in the socioeconomic status of patients as they would be willing to pay a higher amount for HIV treatment if they were gainfully employed or if they had a stable source of income. In addition, the provision of subsidies for low-income earners will ensure improved access to HIV treatment. Although bivariate analysis indicated an association between willingness to pay and income, multivariate regression did not show such an association. The association observed during bivariate analysis was likely influenced by the employment status and not the income on its own.

The role of employment status, level of income, changes in income, and availability of financial support in the willingness and capacity of an individual to pay for health care services cannot be overemphasized.

Patients with a higher level of awareness of the benefits of early initiation of treatment for HIV after diagnosis expressed a higher willingness to pay for such treatment services. This could be because having access to information on the importance of prompt initiation on treatment helps adherence to treatment as this is critical to maintaining viral suppression in patients. Therefore, patients with higher levels of awareness are more likely to explore all opportunities to ensure they remain on treatment even if the services are no longer being provided free of charge.

Further, the perception of the cost of a service or product is likely to be one of the determinants of the willingness to pay for such service or product. A decision on willingness to pay is likely easier if the potential payer is aware of the actual cost. Findings from our study indicated that about 80% of patients perceive the cost of treatment to be higher than what the actual estimated cost is in the public health care facilities. Their perception of the cost was highly exaggerated. This wrong perception is not unexpected as the cost of ARVs and laboratory tests provided in private hospitals and clinics are significantly higher than in the public sector. This is because HIV commodities used in the public sector are procured directly from manufacturers thereby lowering the cost. In addition, the services are not for profit and the personnel cost for the services providers are not usually included in the estimation of the cost of treatment in the public sector. Unlike in the private sector where services are provided for profit, the cost in the public sector is estimated just for cost recovery. While it would be correct to say a willingness to pay for HIV treatment would likely increase if the current cost of treatment is reduced, our study did not find any association between patients' perception of the cost of treatment and willingness to pay for HIV treatment. This finding contradicts other studies which identified the cost of ARVs as a major driver of willingness to pay for HIV treatment services.^28^ A possible reason for this difference is that compared to the early 2010s when the former study was conducted,^28^ the cost of ARVs has reduced significantly in the last fifteen years with as much as a 60% reduction in the cost of generic first-line ARVs in 2019.^29^ Therefore, the cost of ARVs may no longer be a major driver of willingness to pay. However, patients would continue to benefit from a further reduction in the cost of ARVs.

### Limitations

As observed in most studies that employ a contingent valuation approach, the elicitation of willingness to pay can result in response bias. As we highlighted, the expressed willingness to pay and the maximum amount expressed may not equal a capacity to pay or to actual future payments. However, the approach provides an opportunity to gauge the willingness of patients to contribute to their care. In addition, there could be a likelihood of social desirability bias in the responses around income and willingness to pay for treatment. However, we took extra care to word the survey questions to ensure this bias was minimized. Another limitation of the study is that the current willingness to pay did not capture willingness to pay prospectively and future changes in the cost of HIV treatment services. The study did not collect information on the wealth index, so the respondents' economic background reported in this study may not represent a true reflection of the respondents' wealth index.

## CONCLUSION

Patients in Nigeria are willing to contribute to funding for HIV treatment. This can provide some cost recovery through enhanced domestic funding for HIV treatment and equitable access to HIV treatment in Nigeria and similar low-income countries. However, socioeconomic factors play significant roles in willingness and capacity to pay for treatment and the maximum amounts patients are willing to pay. While the cost of accessing HIV treatment continues to decrease in Nigeria and several other countries, additional measures must be put in place to reduce the cost to a level where it becomes affordable to a majority of the patients. This could be complemented by promoting financial empowerment of PLHIV to improve willingness and capacity to pay for treatment and the overall sustainability of funding for HIV treatment in Nigeria and other countries which depend primarily on donor funding for HIV treatment services. Therefore, the government of these countries and donor agencies should consider incorporating income-generating interventions into the HIV program as part of efforts to empower patients to pay for HIV treatment. Future studies should consider using a validated instrument for assessing willingness to pay for health services such as paying for HIV treatment services.

Despite eliciting a higher willingness to pay for HIV treatment services, mechanisms of social health insurance must be explored to ensure the financial protection of those with low capacity to pay. While this study indicated that the funding sustainability of HIV treatment services can be improved through the payment for services by patients, caution must be taken to segment the patients to better target free, subsidized, and full-priced services based on capacity to pay and to ensure equitable access to treatment. While efforts are being made to include HIV treatment into health insurance schemes, the government and other stakeholders must continue to sensitize the public and individuals who are affected by HIV/AIDS of the likelihood of introducing priced HIV treatment services in Nigeria and how they could benefit if they participate in a health insurance scheme rather than paying out-of-pocket. This will require improved transparency around the current funding for HIV treatment services and the opera-tionalization of existing health insurance schemes.

Both bivariate and multivariate analysis indicate that employment status, monthly income, changes in income, awareness of the benefits of the treatment, and availability of financial support influence willingness to pay for treatment services. Program implementers must consider these factors carefully while commercializing HIV treatment services in Nigeria.

## Notes

**Peer Reviewed**

**First published online:** April 6, 2022.
